# Egg-Stage Daily Temperature Amplitude Constrains Adult Fitness and Population Growth in *Tuta absoluta* (Meyrick)

**DOI:** 10.3390/insects17101034

**Published:** 2026-10-09

**Authors:** Xiao-Ning An, Yang Li, Yue Ding, Yun-Wei Liu, Mei-Qi Jia, Da-Qi Li, Wan-Xue Liu, Jian-Yang Guo, Kun Xing

**Affiliations:** 1 Shanxi Agricultural University, Taiyuan 030031, China; 2 Institute of Plant Protection, Chinese Academy of Agricultural Sciences, Beijing 100193, China

**Keywords:** *Tuta absoluta*, daily temperature fluctuation, thermal performance curve, carry-over effects, population growth

## Abstract

Daily temperature variation associated with climate change can strongly affect the survival, development, and population growth of insect species. However, whether the amplitude of daily temperature fluctuation experienced during the early development of insects leads to carry-over effects across the later life stages remains insufficiently understood. Using the invasive pest *Tuta absoluta*, we exposed the eggs to different thermal regimes under two mean-temperature backgrounds (22 and 25 °C) and measured the immediate egg-stage responses and the subsequent carry-over effects. The immediate responses depended on mean temperature, consistent with the nonlinear shape of thermal performance curves. At 22 °C, the wide amplitude accelerated egg development and increased variation in developmental duration without significantly affecting egg survival. At 25 °C, the moderate amplitude accelerated development, whereas the wide amplitude partially reversed this acceleration and produced a steeper age-dependent increase in mortality. Temperature conditions experienced during the egg stage also generated pronounced carry-over effects: wide amplitudes reduced larval survival, shortened female adult longevity, and decreased fecundity, whereas male longevity remained comparatively stable—a pattern consistent with sex-specific sensitivity of adult fitness to early thermal history. The wide amplitude during the egg stage also reduced the intrinsic rate of increase and net reproductive rate. Thus, the thermal regime during the egg stage can suppress population growth through cross-stage effects and sex-dependent fitness responses. Models based only on mean temperature may overestimate population growth potential and mischaracterize phenology. Incorporating egg-stage thermal history and daily temperature fluctuation into phenological and demographic models could improve pest forecasting and integrated pest management decisions under climate change.

## Simple Summary

The tomato leafminer, *Tuta absoluta*, is a destructive invasive pest of tomatoes and other crops. Predictions of its development and population growth are often based only on mean temperature, even though temperatures naturally rise and fall each day. We tested whether daily temperature fluctuations during the egg stage affected both eggs and later life stages. Eggs experienced constant, moderate, or a wide amplitude of temperature fluctuation around two mean temperatures: 22 and 25 °C. After hatching, the insects were reared at the corresponding constant mean temperature. The effects on egg development were dependent on the mean temperature. At 22 °C, the wide amplitude of daily temperature accelerated egg development without significantly reducing egg survival. At 25 °C, the moderate amplitude of daily temperature produced the fastest development, whereas the wide amplitude partly reversed this acceleration. Egg-stage conditions also affected the performance of later stages. The wide amplitude reduced larval survival, shortened female adult life, decreased egg production, and slowed population growth, while male longevity and most pupal traits did not show significant changes. These findings show that temperature variations early in life can influence later reproduction and population growth. Including daily temperature fluctuations and egg-stage thermal history in phenological and demographic models may improve predictions of *T. absoluta* development and outbreak risks and provide more timely pest management tactics.

## 1. Introduction

Global climate change is reshaping the geographic distributions, phenology, and population dynamics of many ectothermic species, including insects [1,2]. In addition to increasing mean temperatures, climate change can alter the amplitude of daily temperature fluctuations because daily maximum and minimum temperatures may change at different rates [3]. Because insects are ectotherms and their body temperatures are closely associated with ambient conditions, their development, survival, metabolism, reproduction, and thermal tolerance can be strongly affected by temperature fluctuations [4,5]. Nevertheless, many insect developmental and phenological models are developed from experiments based on constant temperatures [6], including temperature-dependent models for *T. absoluta* [7]. This approach can overlook the nonlinear relationship between temperature and biological performance described by thermal performance curves (TPCs) [8]. According to Jensen’s inequality, performance under a fluctuating regime is not necessarily equal to the performance predicted from the corresponding mean temperature [9,10,11]. Empirical studies likewise show that daily temperature fluctuations can bring about life history responses that differ from those observed under constant temperatures [12]. Predictive frameworks based on constant or mean temperatures alone may therefore misestimate insect performance in naturally fluctuating thermal environments.

Environmental conditions experienced during early insect development can produce immediate effects in addition to carry-over effects that persist into the later life stages [13,14]. In holometabolous insects, metamorphosis and the functional differentiation of life stages can result in big stage-specific differences in morphology and physiological functioning, potentially buffering against some consequences of the early environmental stress [15,16]. Such modularity in development, however, does not rule out some environmental effects at the early life stages. Adult maintenance and reproductive investment often depend on resources acquired and allocated during pre-adult development [17], so early thermal conditions may influence later fitness through altered developmental trajectories and resource allocation. Despite increasing interest in fluctuating temperatures, it remains unclear whether the thermal conditions at the egg stage affect subsequent larval and pupal performance, adult fitness, and population growth in ways relevant to pest forecasting and risk assessment.

The tomato leafminer, *Tuta absoluta* (Meyrick), is one of the world’s most destructive invasive pests of solanaceous crops [18]. After its detection in Europe in 2006, it rapidly spread through Africa, Asia, and other regions [19]. It poses grave threats to greenhouse and field tomato production and can also damage potato, eggplant, pepper, and other solanaceous crops. Temperature is a major determinant of the survival, development, fecundity, longevity, and population growth [7]. However, most available developmental studies are based on constant temperatures and relatively little is known about how fluctuations in daily temperature affect the development of *T*. *absoluta* under different backgrounds of mean temperatures [20]. The thermal variability at the egg stage can generate cross-stage effects in small ectotherms [21], but whether such effects persist through the larval, pupal, and adult stages of *T. absoluta* remains unexplored. Detailed studies are therefore needed to improve phenological forecasting, invasion-risk assessment, and integrated pest management under climate change [18,22].

In this study, we established different thermal regimes under two backgrounds of mean temperatures: 22 °C and 25 °C. Based on greenhouse observations, the following regimes were adopted: 22 ± 0, 22 ± 6, and 22 ± 12 °C; and 25 ± 0, 25 ± 5, and 25 ± 10 °C. Eggs of *T*. *absoluta* were exposed to these regimes, after which hatched larvae were reared at the corresponding constant mean temperatures. This design separated immediate egg-stage responses from carry-over effects on subsequent life-history traits. We asked whether (1) the effects of temperature amplitude on egg development and survival depend on mean temperature, as predicted by nonlinear TPC theory; (2) egg-stage temperature amplitude generates carry-over effects on subsequent developmental stages and descriptively different responses in female and male adult fitness; and (3) egg-stage temperature amplitude alters population growth in ways relevant to pest forecasting and integrated pest management.

## 2. Materials and Methods

### 2.1. Host Plants

Tomato seeds (cultivar: Maofen 802; Xi’an Shenzhou Seed Industry Co., Ltd., Xi’an, China) were soaked in sterile water for 24 h at a laboratory ambient temperature of 20–25 °C, maintained by central air conditioning, and the water was replenished every 8 h to ensure full seed hydration. Imbibed seeds were sown into plastic pots (11 cm × 8 cm × 11 cm; top diameter × bottom diameter × height) filled with sterilized potting soil, with five to seven seeds planted per pot. Seeds were covered with a 0.5 cm layer of sterilized soil and gently pressed. The pots were arranged in plastic trays (35 cm × 50 cm × 5 cm; length × width × depth). Bottom watering was used to avoid displacing seedlings; specifically, 1 cm of sterile water was added to each tray to maintain substrate moisture. Plants were maintained in an artificial climate chamber (RXZ-380-LED, Ningbo Jiangnan Instrument Factory, Ningbo, China) at 22 ± 1 °C, 50–70% relative humidity (RH), and a 16:8 h light:dark photoperiod (light from 05:00 to 21:00; dark from 21:00 to 05:00) under full-spectrum LED lights (42,000 lux). Substrate moisture was checked every other day, and sterile water was added to maintain the 1 cm water depth. When plants reached the 7–9 true-leaf stage, healthy, uniform, and pest-free individuals were selected for *T. absoluta* rearing and experiments.

### 2.2. Test Insects

A laboratory colony of *T*. *absoluta* was established from individuals collected on 3 May 2020 in a greenhouse tomato field (cultivar: Maofen 802; Xi’an Shenzhou Seed Industry Co., Ltd., Xi’an, China) in Baiban Township, Jiancaoping District, Taiyuan City, Shanxi Province, China (38.04° N, 112.47° E). Curled tomato leaves containing pupae were transferred to pots, each containing six healthy tomato plants at the 7–9 true-leaf stage, and the pots were placed in ventilated transparent rearing cages (55 cm × 35 cm × 35 cm; length × width × depth). To maintain colony consistency, the mating procedure was standardized each generation. Uniformly sized adults emerging on the same day were selected for mating. Approximately 200 fresh, plump eggs laid on the first day after mating were transferred to new cages containing six healthy tomato plants per pot. The colony was maintained at 22 ± 1 °C, 50–70% RH, and a 16:8 h light:dark photoperiod (light from 05:00 to 21:00; dark from 21:00 to 05:00) under full-spectrum LED lights (42,000 lux). Insects were reared continuously for 10 generations before the experiments to establish a stable laboratory population under standardized conditions. Similar multi-generation laboratory rearing has been used in previous studies of *T. absoluta* [23,24].

### 2.3. Temperature Design

Real-time temperatures were monitored in a greenhouse tomato field in Baiban Township during May 2021 and 2022 using a data logger (U23-001, HOBO Ltd., Bourne, MA, USA). The daily mean temperature was approximately 22 °C (Figure S1A), and the maximum amplitude of daily temperature was approximately ±12 °C (Figure S1B). The temperature amplitude was defined as A = (Tmax − Tmin)/2, i.e., the deviation of the daily maximum or minimum from the daily mean. Thus, at a mean temperature of 22 °C, an amplitude of ±12 °C corresponds to approximate daily temperature extremes of 10 and 34 °C. The 22 °C treatment was based on the observed greenhouse mean temperature, while 25 °C was set 3 °C higher to represent a warmer thermal background. The wide fluctuation treatments approximated the upper range observed in the greenhouse, and intermediate amplitudes were included to establish different thermal regimes. Temperature regimes were as follows: 22 ± 0, 22 ± 6 (moderate amplitude), 22 ± 12 (wide amplitude), 25 ± 0, 25 ± 5 (moderate amplitude), and 25 ± 10 (wide amplitude)°C. The 22 °C treatment represented the current greenhouse thermal background based on the observed mean temperature, whereas the 25 °C treatment was set 3 °C higher as an experimental warmer background in the context of climate warming [3]. The +3 °C increment was used as an experimental warming contrast rather than as a direct projection of a specific future climate scenario. During egg exposure, fluctuating treatments followed a continuous 24 h daily temperature cycle rather than stepwise changes at 6- or 12 h intervals. Temperature gradually increased from the daily minimum to the daytime maximum and then decreased to the minimum again (Figure S2A,B). The experiment was conducted in three independent temporal blocks. Within each block, the six temperature regimes were randomly assigned to the available artificial climate chambers. Temperature assignments were rotated among chambers between blocks so that no temperature regime was consistently associated with a particular chamber. Each chamber–block combination constituted an independent experimental unit. Actual temperature and relative humidity were recorded every 15 min in each chamber using a data logger. Experimental block and chamber identity were included as random effects in the statistical analyses. Full-spectrum LED lights (42,000 lux) provided a 16:8 h light:dark photoperiod (light from 05:00 to 21:00; dark from 21:00 to 05:00) in all chambers. The same 50–70% RH range was also maintained in all chambers, and RH was not manipulated as an experimental factor [24,25]. Laboratory temperature was regulated at 22 ± 1 °C using central air conditioning to minimize external interference with chamber operation. Recorded temperatures closely matched the target regimes throughout the experiment (Figure S2C,D).

### 2.4. Experimental Procedure

Uniform, *T. absoluta* eggs were gently collected from tomato leaves using a sterile fine brush moistened with sterile water. Eggs were laid during a 4 h oviposition period on the first day of adult pairing. Eggs were divided into batches of 30–40, and each batch was evenly distributed onto 3–4 fresh tomato leaves. The leaves were placed in sterile Petri dishes (9 cm diameter) lined with sterile filter paper saturated with sterile water with dripping. Dishes were placed into artificial climate chambers set to the different temperature-amplitude regimes. A total of 707 eggs were assigned to treatments as follows: 22 ± 0 °C (*n* = 114), 22 ± 6 °C (*n* = 115), 22 ± 12 °C (*n* = 168), 25 ± 0 °C (*n* = 107), 25 ± 5 °C (*n* = 98), and 25 ± 10 °C (*n* = 105). Here, *n* represents the number of individual eggs assigned to each temperature treatment, not the number of egg batches or Petri dishes. The unequal sample sizes resulted from variation in the number of viable eggs available from the oviposition batches at the time of treatment assignment; all available eggs meeting the experimental criteria were used rather than discarding eggs to force equal sample sizes among treatments. Artificial climate chambers were maintained at 50–70% RH and a 16:8 h light:dark photoperiod (light from 05:00 to 21:00; dark from 21:00 to 05:00) under full-spectrum LED lights (42,000 lux). Egg hatching was checked daily at 08:00. Eggs that turned black or brown were recorded as dead. Immediately after hatching, larvae from all treatment groups were transferred to the corresponding constant mean temperatures (22 °C or 25 °C) for subsequent rearing. Sterile water was added daily to the filter paper at 08:00 using a dropper to keep the leaves moist. When leaves wilted or larval mining damaged approximately 50% of the leaf area, fresh leaves of similar size were provided to ensure sufficient and consistent food supply throughout larval development. Larvae were recorded as dead if their bodies became stiff and turned black or dark brown. Pupation started when larvae became spindle-shaped and spun a thin silken layer. Pupae were individually transferred to transparent glass tubes (1.5 cm × 8 cm; bottom diameter × height), which were plugged with sterile cotton to prevent adult escape. Adult emergence was monitored daily at 08:00 and 20:00. After emergence, healthy, uniformly sized, newly emerged adults (<12 h old) from the same treatment were paired at a 1:1 female:male ratio. Each pair was placed in a new tube (1.5 cm × 8 cm; bottom diameter × height). A cotton ball soaked in 10% honey solution was provided for nutrition, and a fresh tomato leaf piece (5 cm × 1 cm; length × width) was added as an oviposition substrate. The leaf piece and honey-soaked cotton were replaced daily at 08:00. The daily number of eggs laid on the leaf and inner tube wall was recorded until both adults died. Development, survival, longevity, and fecundity were recorded daily at 08:00 until all tested individuals died. Developmental duration was defined as the time spent in each developmental stage (egg, larva, and pupa). Adult longevity was defined as the period from emergence to death, and fecundity was defined as the total number of eggs laid per female.

### 2.5. Statistical Analysis

Normality and homogeneity of variance were evaluated using Shapiro–Wilk and Levene’s tests, respectively. Most response variables were continuous, positive, and right-skewed. Therefore, generalized linear models (GLMs) with a Gamma distribution were used. Analyses were conducted separately within the 22 and 25 °C mean-temperature backgrounds. Response variables included egg, larval, female pupal, and male pupal developmental duration, adult longevity, fecundity, and their coefficients of variation (CVs), which were calculated following [26]. CV was used rather than standard deviation to compare relative among-individual variability because the mean values of life-history traits differed among temperature treatments. By standardizing dispersion relative to the mean, CV facilitates comparisons among treatments with different trait means. The 95% confidence intervals for CV were reported in Table S3. Pairwise comparisons of estimated marginal means were conducted with Holm–Bonferroni adjustment for multiple comparisons [27]. Survival was analyzed using Kaplan–Meier curves and log-rank tests [28]. Mortality dynamics were further characterized using a two-parameter Weibull model, S(t) = exp[−(t/η)^β^], where S(t) is the probability of surviving beyond time t, η is the scale parameter, and β is the shape parameter [29]. The scale parameter η represents the characteristic time scale of mortality, whereas β describes the temporal pattern of mortality risk. These parameters were used to compare differences in the timing and age dependence of mortality among temperature treatments [30]. Population parameters were calculated using TWOSEX-MSChart [31], including mean generation time (*T*), net reproductive rate (*R*_0_), intrinsic rate of increase (*r_m_*), and finite rate of increase (*λ*). Net reproductive rate was calculated as *R*_0_ = ∑*l_x_m_x_*, representing the expected number of offspring produced by an individual over its lifetime. The intrinsic rate of increase rm was obtained from the Euler–Lotka equation ∑e*^−r^_m_*^(*x*+1)^*l_x_m_x_* = 1, and represents the potential instantaneous rate of population increase under the specified environmental conditions. The finite rate of increase was calculated as *λ* = e*^r^_m_*, representing the multiplicative population growth rate per unit time. Mean generation time was calculated as *T* = ln (*R*_0_)/*r_m_*, representing the average time required for the population to increase by a factor of *R*_0_. These demographic parameters were selected because together they describe complementary aspects of population performance, including reproductive output, generation turnover, and potential population growth. Standard errors of these parameters were estimated using nonparametric bootstrap resampling with 1000 replicates. Differences among temperature amplitude treatments within each mean temperature background were evaluated using paired bootstrap tests implemented in TWOSEX-MSChart. Statistical significance was determined at *p* < 0.05. All population parameters are presented as the bootstrap estimate ± bootstrap standard errors [32,33]. Statistical analyses were conducted using IBM SPSS Statistics for Windows, version 26.0 (IBM Corp., Armonk, NY, USA), and figures were produced using Origin 2021 (OriginLab Corp., Northampton, MA, USA).

## 3. Results

### 3.1. Immediate Effects of Temperature Amplitude on Egg Development and Survival

**Figure 1:**
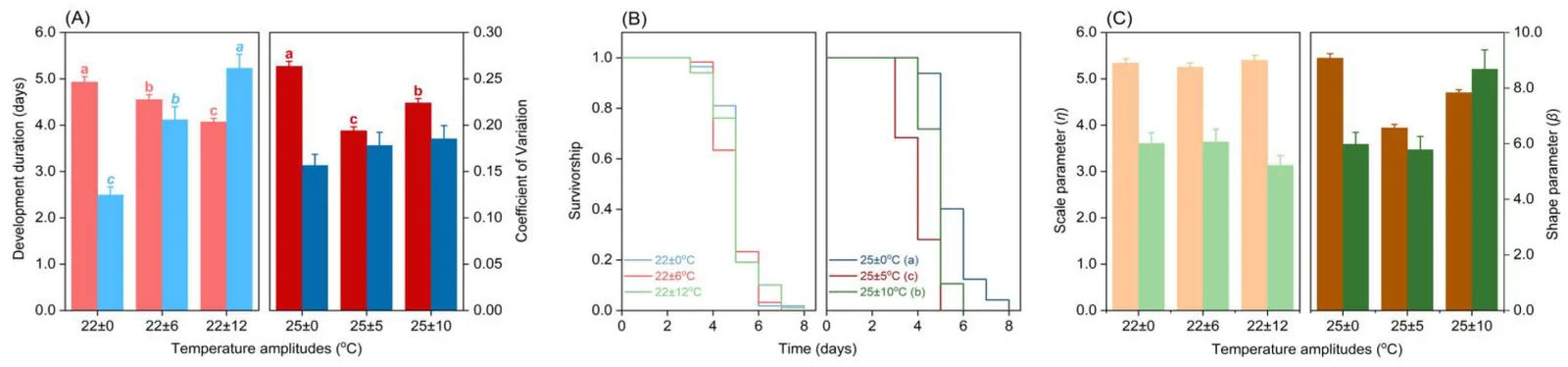
Immediate effects of egg-stage temperature amplitude on developmental duration and its coefficient of variation (**A**), survivorship (**B**), and Weibull mortality parameters (**C**) of *Tuta absoluta* eggs. In (**A**), red bars represent developmental duration (left *y*-axis), and blue bars represent the coefficient of variation (right *y*-axis). Different lowercase letters within each metric indicate significant differences among treatments (*p* < 0.05); the absence of letters indicates no significant difference. In (**B**), different lowercase letters next to the legends indicate significant differences among survival curves (log-rank test, *p* < 0.05). In (**C**), orange bars represent the Weibull scale parameter (*η*, characteristic life), and green bars represent the shape parameter (*β*, temporal change in mortality hazard). Bar-chart data are presented as mean ± SE. Sample sizes were 114, 115, 168, 107, 98, and 105 eggs for the 22 ± 0, 22 ± 6, 22 ± 12, 25 ± 0, 25 ± 5, and 25 ± 10 °C treatments, respectively.

At 22 °C, egg developmental duration significantly decreased with increasing temperature amplitude (χ^2^ = 43.080, df = 2, *n* = 299, *p* < 0.001). Compared with the constant-temperature treatment (22 ± 0 °C; 4.93 d), developmental duration at 22 ± 6 °C and 22 ± 12 °C was shortened by 0.38 d and 0.86 d, respectively. The coefficient of variation (CV) for egg developmental duration also increased significantly with the wide amplitude (χ^2^ = 71.777, df = 2, *n* = 299, *p* < 0.001). At 25 °C, temperature amplitude significantly affected egg developmental duration (χ^2^ = 109.063, *df* = 2, *n* = 274, *p* < 0.001). Developmental duration was the shortest at 25 ± 5 °C (3.88 d), which was significantly lower than that at 25 ± 0 °C (5.27 d) and 25 ± 10 °C (4.48 d). The CV for egg developmental duration did not differ significantly among treatments (χ^2^ = 2.695, df = 2, *n* = 274, *p* = 0.260) (Figure 1A; Table S1).

Egg survival at 22 °C was not significantly affected by temperature amplitude (χ^2^ = 1.554, df = 2, *n* = 397, *p* = 0.459). The Weibull scale parameter (η) was similar among treatments (5.25–5.40), whereas the shape parameter (β) decreased slightly as amplitude increased (22 ± 0 °C: 6.01; 22 ± 6 °C: 6.07; 22 ± 12 °C: 5.23). In contrast, at 25 °C, egg survival was significantly higher at 25 ± 5 °C than at 25 ± 0 °C or 25 ± 10 °C (χ^2^ = 137.605, df = 2, *n* = 310, *p* < 0.001). The scale parameter showed a non-linear response to amplitude (25 ± 0 °C: 5.90; 25 ± 5 °C: 4.27; 25 ± 10 °C: 5.09), and the shape parameter peaked at 25 ± 10 °C (8.69), and was numerically higher than values at 25 ± 0 °C (5.99) and 25 ± 5 °C (5.79) (Figure 1B,C; Table S2).

### 3.2. Carry-Over Effects of Temperature Amplitude on Larval Development and Survival

**Figure 2:**
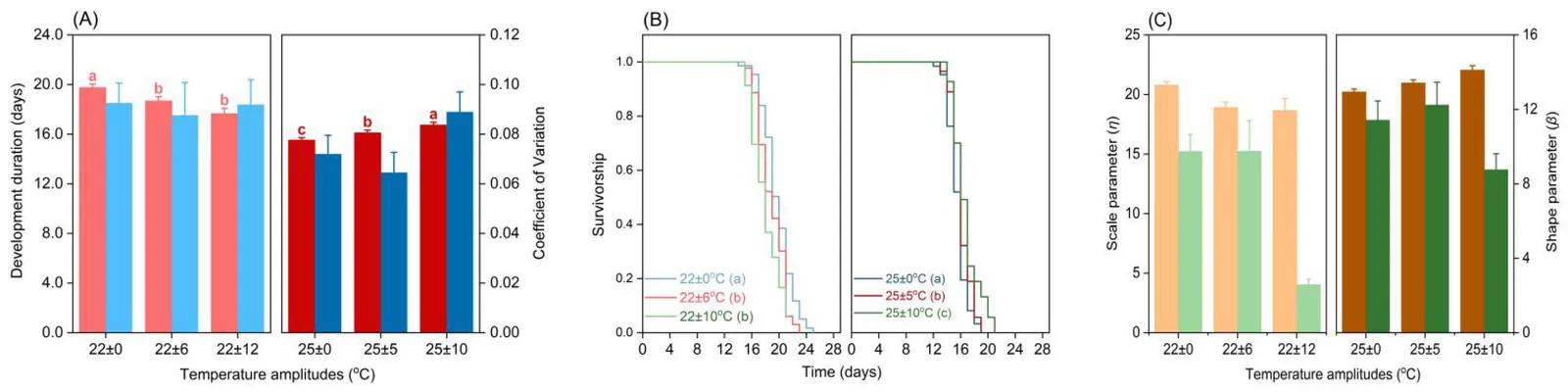
Carry-over effects of egg-stage temperature amplitude on developmental duration and its coefficient of variation (**A**), survivorship (**B**), and Weibull mortality parameters (**C**) of *Tuta absoluta* larvae. In (**A**), red bars represent developmental duration (left *y*-axis), and blue bars represent the coefficient of variation (right *y*-axis). Different lowercase letters within each metric indicate significant differences among treatments (*p* < 0.05); the absence of letters indicates no significant difference. In (**B**), different lowercase letters next to the legends indicate significant differences among survival curves (log-rank test, *p* < 0.05). In (**C**), orange bars represent the Weibull scale parameter (*η*, characteristic life), and green bars represent the shape parameter (*β*, temporal change in mortality hazard). Bar-chart data are presented as mean ± SE. Sample sizes for larvae were 110, 95, 94, 97, 92, and 85 for the 22 ± 0, 22 ± 6, 22 ± 12, 25 ± 0, 25 ± 5, and 25 ± 10 °C treatments, respectively.

At 22 °C, increasing temperature amplitude significantly shortened larval developmental duration (χ^2^ = 17.628, df = 2, *n* = 118, *p* < 0.001). Compared with 22 ± 0 °C (19.75 d), larval developmental duration at 22 ± 6 °C and 22 ± 12 °C was shortened by 1.07 d and 2.10 d, respectively. Temperature amplitude had no significant effect on the CV for larval developmental duration (χ^2^ = 0.101, df = 2, *n* = 118, *p* = 0.951). At 25 °C, larval developmental duration increased significantly with wide amplitude (χ^2^ = 17.717, df = 2, *n* = 168, *p* < 0.001). Developmental durations at 25 ± 0 °C, 25 ± 5 °C, and 25 ± 10 °C were 15.52, 16.11, and 16.74 d, respectively. Similar to the 22 °C group, the CV for larval developmental duration was not significantly affected by temperature amplitude (χ^2^ = 4.766, df = 2, *n* = 168, *p* = 0.092) (Figure 2A; Table S1).

Larval survival at 22 °C was significantly higher in the 22 ± 0 °C treatment than in the 22 ± 6 °C and 22 ± 12 °C treatments (χ^2^ = 10.337, df = 2, *n* = 299, *p* = 0.006). The scale parameter tended to decrease with increasing amplitude (22 ± 0 °C: 20.79; 22 ± 6 °C: 18.91; 22 ± 12 °C: 18.65), and the shape parameter at 22 ± 12 °C (2.58) was lower than that at 22 ± 0 °C (9.74) and 22 ± 6 °C (9.76). At 25 °C, increasing temperature amplitude significantly reduced larval survival (χ^2^ = 16.385, df = 2, *n* = 274, *p* < 0.001). The scale parameter increased with wide amplitude (25 ± 0 °C: 16.17; 25 ± 5 °C: 16.78; 25 ± 10 °C: 17.64), whereas the shape parameter tended to decrease (25 ± 0 °C: 11.42; 25 ± 5 °C: 12.24; 25 ± 10 °C: 8.76) (Figure 2B,C; Table S2).

### 3.3. Carry-Over Effects of Temperature Amplitude on Pupal Development and Survival

**Figure 3:**
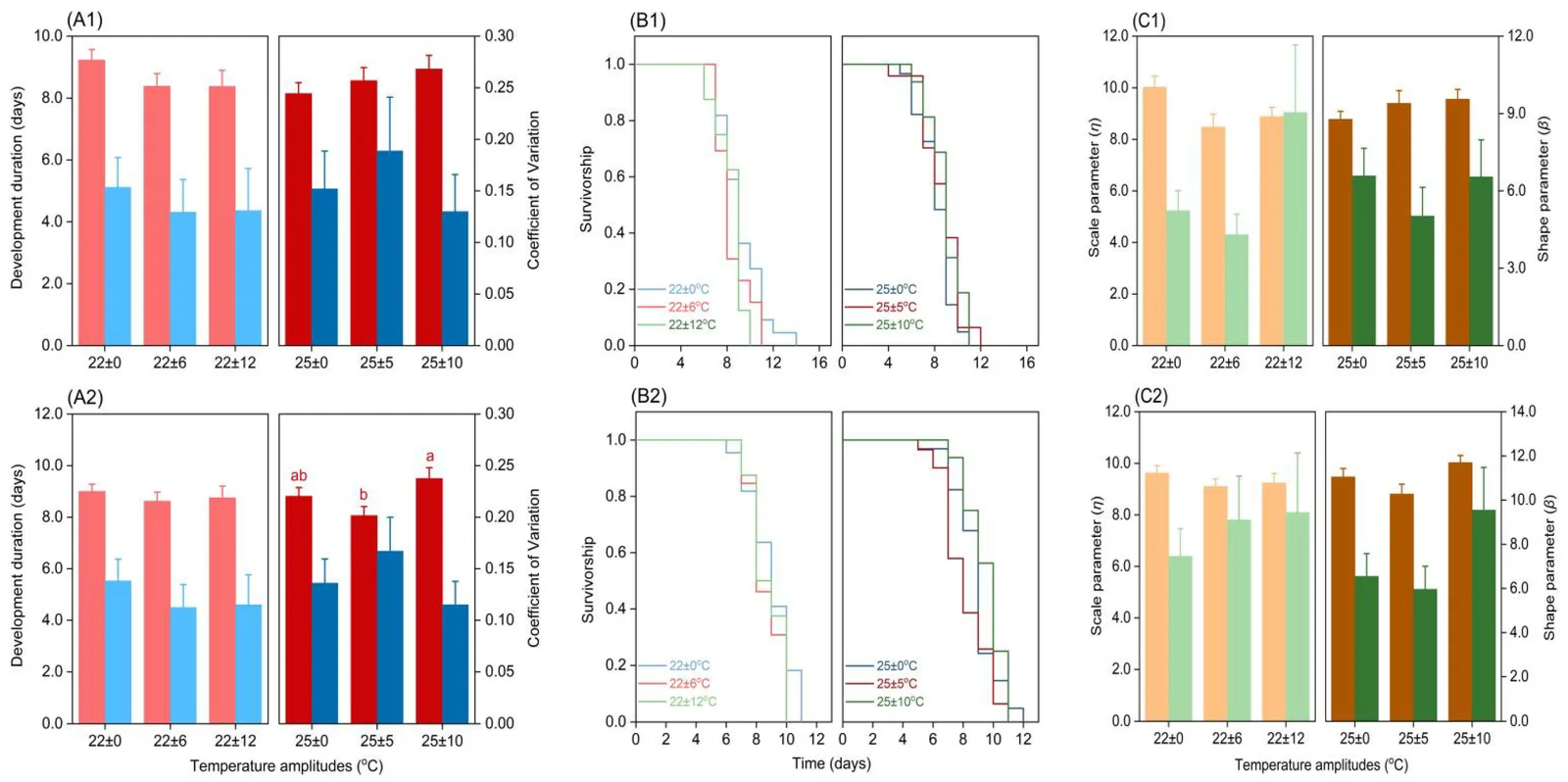
Carry-over effects of egg-stage temperature amplitude on developmental duration and its coefficient of variation ((**A1**), Female pupae; (**A2**), male pupae), survivorship ((**B1**), Survivorship curves for female pupae; (**B2**), survivorship curves for male pupae), and Weibull mortality parameters ((**C1**), Female pupae; (**C2**), male pupae) of *Tuta absoluta*. In (**A**), red bars represent developmental duration (left *y*-axis), and blue bars represent the coefficient of variation (right *y*-axis). Different lowercase letters within each metric indicate significant differences among treatments (*p* < 0.05); the absence of letters indicates no significant difference. In (**B**), different lowercase letters next to the legends indicate significant differences among survival curves (log-rank test, *p* < 0.05). In (**C**), orange bars represent the Weibull scale parameter (*η*, characteristic life), and green bars represent the shape parameter (*β*, temporal change in mortality hazard). Bar-chart data are presented as mean ± SE. Sample sizes for female pupae were 29, 21, 10, 30, 24, and 29 for the 22 ± 0, 22 ± 6, 22 ± 12, 25 ± 0, 25 ± 5, and 25 ± 10 °C treatments, respectively, and sample sizes for male pupae were 31, 17, 10, 32, 29, and 24 for the 22 ± 0, 22 ± 6, 22 ± 12, 25 ± 0, 25 ± 5, and 25 ± 10 °C treatments, respectively.

At 22 °C, temperature amplitudes had no significant carry-over effects on female pupal developmental duration (χ^2^ = 3.256, df = 2, *n* = 43, *p* = 0.196) or its CV (χ^2^ = 0.386, df = 2, *n* = 43, *p* = 0.824). Similarly, at 25 °C, temperature amplitudes had no significant carry-over effects on either female pupal developmental duration (χ^2^ = 2.044, df = 2, *n* = 53, *p* = 0.360) or its CV (χ^2^ = 0.927, df = 2, *n* = 53, *p* = 0.629) (Figure 3(A1); Table S1).

Regarding female pupal survival, temperature amplitudes showed no significant carry-over effects at 22 °C (χ^2^ = 2.947, df = 2, *n* = 60, *p* = 0.229). The scale parameter (η) decreased slightly as temperature amplitudes increased (22 ± 0 °C: 10.02; 22 ± 6 °C: 8.47; 22 ± 12 °C: 8.88). The shape parameter (β) at 22 ± 12 °C (9.04) was numerically higher than values at 22 ± 0 °C (5.23) and 22 ± 6 °C (4.30). At 25 °C, female pupal survival was also not significantly affected by temperature amplitudes (χ^2^ = 2.702, df = 2, *p* = 0.259). The scale parameter (η) increased slightly with wide amplitudes (25 ± 0 °C: 8.78; 25 ± 5 °C: 9.40; 25 ± 10 °C: 9.56). Meanwhile, the shape parameter (β) exhibited a non-linear response to increasing temperature amplitudes (25 ± 0 °C: 6.58; 25 ± 5 °C: 5.03; 25 ± 10 °C: 6.55) (Figure 3(B1,C1); Table S2).

At 22 °C, temperature amplitude had no significant carry-over effects on male pupal developmental duration (χ^2^ = 0.764, df = 2, *n* = 43, *p* = 0.683) or its CV (χ^2^ = 0.844, df = 2, *n* = 43, *p* = 0.656). At 25 °C, temperature amplitude significantly affected male pupal developmental duration (χ^2^ = 7.075, df = 2, *n* = 53, *p* = 0.029), but did not significantly affect its CV (χ^2^ = 1.817, df = 2, *n* = 53, *p* = 0.403). Developmental durations at 25 ± 0 °C, 25 ± 5 °C, and 25 ± 10 °C were 8.81, 8.06, and 9.50 d, respectively (Figure 3(A2); Table S1).

Regarding male pupal survival at 22 °C, temperature amplitudes showed no significant carry-over effects (χ^2^ = 1.549, df = 2, *n* = 58, *p* = 0.461). The scale parameters (η) were similar among treatments (22 ± 0 °C: 9.62; 22 ± 6 °C: 9.10; 22 ± 12 °C: 9.24). However, the shape parameter (β) trended upward as temperature amplitudes increased (22 ± 0 °C: 7.46, 22 ± 6 °C: 9.11, 22 ± 12 °C: 9.45). At 25 °C, male pupal survival was also not significantly affected by treatments (χ^2^ = 5.180, df = 2, *n* = 85, *p* = 0.075). The scale parameter (η) reached its maximum at 25 ± 10 °C (25 ± 0 °C: 9.47; 25 ± 5 °C: 8.81; 25 ± 10 °C: 10.03). The shape parameter (β) at 25 ± 10 °C (9.55) was numerically higher than values at 25 ± 0 °C (6.56) and 25 ± 5 °C (5.97) (Figure 3(B2,C2); Table S2).

### 3.4. Carry-Over Effects of Temperature Amplitude on Adult Longevity

**Figure 4:**
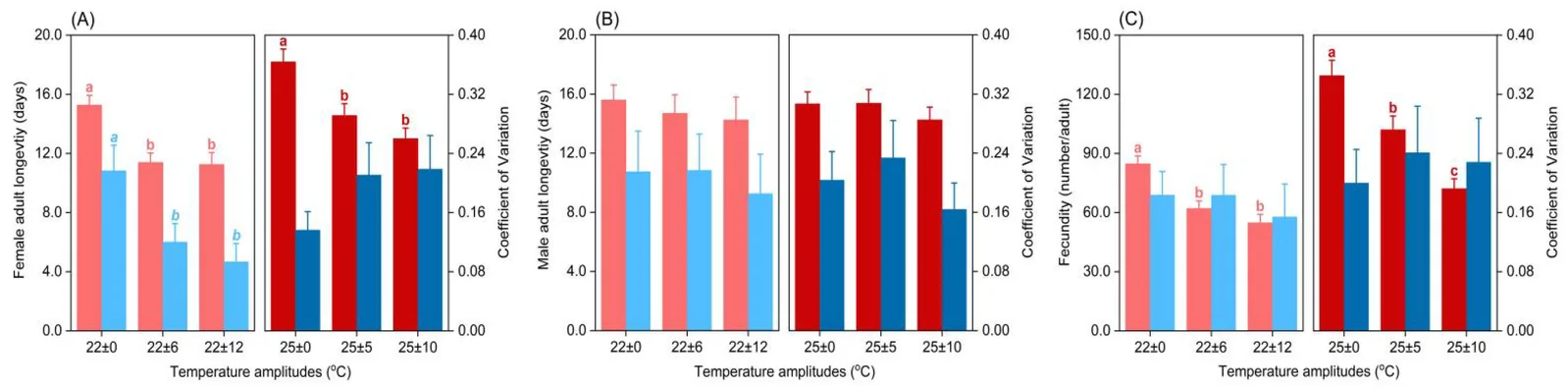
Carry-over effects of egg-stage temperature amplitude on longevity and its coefficient of variation in *Tuta absoluta* females (**A**) and males (**B**), and on fecundity and its coefficient of variation (**C**). Red bars represent longevity or fecundity (left *y*-axis), and blue bars represent the coefficient of variation (right *y*-axis). Different lowercase letters within each metric indicate significant differences among treatments (*p* < 0.05); the absence of letters indicates no significant difference. Bar-chart data are presented as mean ± SE. Sample sizes were the same for adult females and males: 22, 13, 8, 21, 16, and 16 per sex for the 22 ± 0, 22 ± 6, 22 ± 12, 25 ± 0, 25 ± 5, and 25 ± 10 °C treatments, respectively.

At 22 °C, female longevity significantly shortened as temperature amplitude increased (χ^2^ = 23.496, df = 2, *n* = 43, *p* < 0.001). Compared with the 22 ± 0 °C treatment (15.27 d), longevity at 22 ± 6 °C and 22 ± 12 °C decreased by 3.89 d and 4.02 d, respectively. The CV for female longevity also decreased significantly with wide amplitude (χ^2^ = 9.665, df = 2, *n* = 43, *p* = 0.008). At 25 °C, female longevity also decreased significantly with amplitude (χ^2^ = 22.874, df = 2, *n* = 53, *p* < 0.001). Longevity at 25 ± 0 °C, 25 ± 5 °C, and 25 ± 10 °C was 18.19, 14.56, and 13.00 d, respectively. However, the CV at 25 °C was not significantly affected (χ^2^ = 3.810, df = 2, *n* = 53, *p* = 0.149) (Figure 4A; Table S1). By contrast, temperature amplitude had no significant carry-over effects on male longevity or its CV at either 22 °C (longevity: χ^2^ = 0.625, df = 2, *n* = 43, *p* = 0.732; CV: χ^2^ = 3.239, df = 2, *n* = 43, *p* = 0.198) or 25 °C (longevity: χ^2^ = 1.061, df = 2, *n* = 53, *p* = 0.588; CV: χ^2^ = 1.362, df = 2, *n* = 53, *p* = 0.506) (Figure 4B; Table S1).

### 3.5. Carry-Over Effects of Temperature Amplitude on Fecundity

At 22 °C, wide amplitudes significantly reduced fecundity (χ^2^ = 29.017, df = 2, *n* = 43, *p* < 0.001). Compared with the 22 ± 0 °C treatment (84.68 eggs/female), fecundity at 22 ± 6 °C and 22 ± 12 °C decreased to 62.08 and 54.75 eggs/female, respectively. The CV for fecundity was not significantly affected (χ^2^ = 0.301, df = 2, *n* = 43, *p* = 0.860). At 25 °C, fecundity also declined significantly as amplitude increased (χ^2^ = 41.165, df = 2, *n* = 53, *p* < 0.001). Total eggs laid at 25 ± 0 °C, 25 ± 5 °C, and 25 ± 10 °C were 129.43, 102.00, and 72.13 eggs/female, respectively. Similar to the 22 °C group, the CV was not significantly affected (χ^2^ = 0.319, df = 2, *n* = 53, *p* = 0.853) (Figure 4C; Table S1).

### 3.6. Carry-Over Effects of Temperature Amplitude on Population Parameters

**Figure 5:**
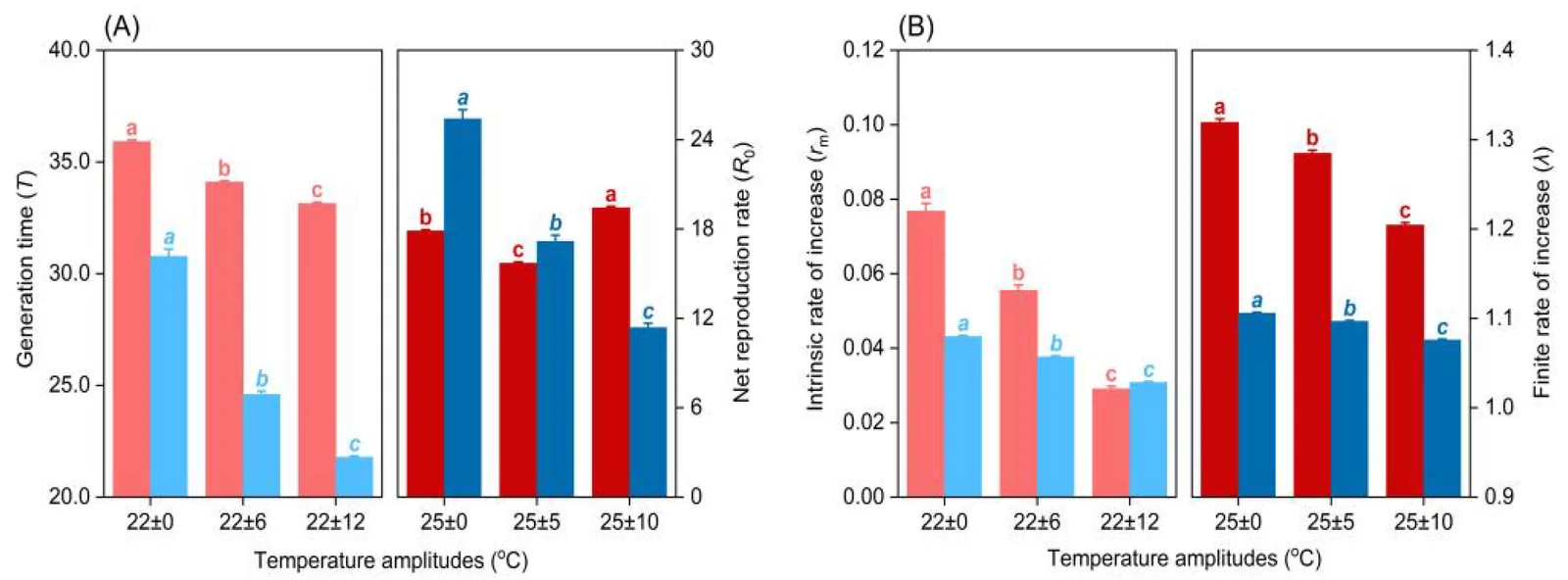
Carry-over effects of egg-stage temperature amplitude on population parameters of female (**A**) and male (**B**) *Tuta absoluta*. In (**A**), red bars represent generation time (*T*; left *y*-axis), and blue bars represent net reproductive rate (*R*_0_; right *y*-axis). In (**B**), red bars represent intrinsic rate of increase (*r_m_*; left *y*-axis), and blue bars represent finite rate of increase (*λ*; right *y*-axis). Different lowercase letters above bars indicate significant differences among treatments based on paired bootstrap tests (*p* < 0.05). Bar-chart data are presented as mean ± SE.

At 22 °C, temperature amplitude significantly affected generation time (*T*) (*p* < 0.001). *T* shortened from 35.91 ± 0.07 d at 22 ± 0 °C to 33.13 ± 0.07 d at 22 ± 12 °C. Net reproductive rate (*R*_0_) also decreased significantly (*p* < 0.001). Compared with 22 ± 0 °C (16.17 ± 0.48), *R*_0_ at 22 ± 6 °C and 22 ± 12 °C decreased to 6.91 ± 0.20 and 2.68 ± 0.08, respectively. At 25 °C, temperature amplitude also significantly affected *T* (*p* < 0.001). Compared with 25 ± 0 °C (31.91 ± 0.06 d), *T* was shortened at 25 ± 5 °C (30.46 ± 0.06 d) but prolonged at 25 ± 10 °C (32.94 ± 0.07 d). Furthermore, *R*_0_ decreased significantly with wide amplitude (*p* < 0.001). Compared with 25 ± 0 °C (25.41 ± 0.61), *R*_0_ at 25 ± 5 °C (17.17 ± 0.41) and 25 ± 10 °C (11.39 ± 0.27) decreased by 32.4% and 55.2%, respectively (Figure 5A).

The intrinsic rate of increase (*r_m_*) decreased significantly with wide amplitude at both 22 °C (*p* < 0.001) and 25 °C *p* < 0.001). At 22 °C, *r_m_* values for 22 ± 0 °C, 22 ± 6 °C, and 22 ± 12 °C were 0.0768 ± 0.0020, 0.0555 ± 0.0015, and 0.0290 ± 0.0008, respectively. At 25 °C, *r_m_* values for 25 ± 0 °C, 25 ± 5 °C, and 25 ± 10 °C were 0.1006 ± 0.0010, 0.0923 ± 0.0009, and 0.0730 ± 0.0007, respectively. Finally, the finite rate of increase (*λ*) showed a significant downward trend with increasing amplitude at both 22 °C (*p* < 0.001) and 25 °C (*p* < 0.001) (Figure 5B).

## 4. Discussion

### 4.1. Mean-Temperature Background Shaped Immediate Egg-Stage Thermal Amplitude Effects

The immediate effects of thermal treatments on egg development and survival depended strongly on the mean-temperature background rather than representing a simple linear sum of high- and low-temperature effects. This pattern is consistent with thermal performance theory and Jensen’s inequality, which predict that the response to fluctuation depends on the position of the mean temperature along the TPC [10,11,34]. At 22 °C, the wide amplitude (22 ± 12 °C) shortened mean egg developmental duration from 4.93 to 4.07 d. A plausible interpretation is that the acceleration produced during warmer portions of the cycle outweighed inhibition during cooler portions [5,35,36]. The concurrent increase in the CV of egg developmental duration indicates greater among-individual variation in developmental timing. This greater variation suggests reduced hatching synchrony but does not by itself identify the underlying developmental mechanism. At 25 °C, the response was nonlinear: 25 ± 5 °C produced the shortest development, whereas 25 ± 10 °C partially reversed that acceleration, although development remained faster than at 25 ± 0 °C. The Weibull shape parameter was numerically higher at 25 ± 10 °C than at the other 25 °C treatments, indicating a steeper age-dependent increase in mortality hazard rather than, by itself, a higher absolute mortality probability. The 35 °C daily maximum under 25 ± 10 °C lies at the upper end of the temperature range examined for *T. absoluta* development and above the range associated with optimal immature performance [7,37], which may explain the partial loss of developmental acceleration. Overall, models based only on mean temperature may misestimate developmental timing, synchrony, and survival under fluctuating thermal conditions. The present study focused on life-history and demographic responses and did not include histological, physiological, or molecular measurements. Therefore, the mechanisms underlying the effects of thermal conditions on egg survival remain unclear and warrant further investigation.

### 4.2. Egg-Stage Thermal History Generated Stage-Specific Carry-Over Effects

Egg-stage temperature amplitude generated clear carry-over effects on larval performance, and the direction of the developmental response depended on mean temperature. At 22 °C, wide egg-stage amplitudes shortened subsequent larval development, whereas larval survival declined and the Weibull shape parameter was numerically lower at the wide amplitude. Faster development therefore co-occurred with lower survival, producing a fast-development and low-survival pattern. At 25 °C, wide egg-stage amplitudes prolonged larval development and reduced survival, producing a delayed development and low survival pattern. These contrasting responses show that carry-over effects cannot be generalized independently of mean-temperature background. Most pupal endpoints showed no detectable response to egg-stage temperature amplitude, suggesting that sensitivity to early thermal history varied among life stages. Compared with larval traits, pupal traits appeared less sensitive to egg-stage thermal history, indicating substantial stage-specific differences in sensitivity to early temperature variation in *T. absoluta*. This stage-specific response may be associated with a functional differentiation across the complex life cycle of holometabolous insects. As a key period of morphological reorganization and energy redistribution, the pupal stage may partially buffer against the adverse effects of early thermal stress on certain developmental traits [38].

### 4.3. Female Adult Fitness Was More Sensitive than Male Longevity

Regardless of mean temperature, wide amplitudes during the egg stage significantly reduced female adult longevity and fecundity, indicating that the adverse effects of early thermal history persisted into adulthood. These findings do not fully support a strict interpretation of life-cycle modularity, in which metamorphosis may buffer against early environmental stress through strong functional differentiation among developmental stages [16]. In the present study, although pupal developmental duration and survival were unaffected, the negative effects of egg-stage temperature amplitude remained evident in female longevity and reproductive output, suggesting that the ecological consequences of early thermal stress were not completely eliminated during metamorphosis [13,39]. Overall, most CVs were not significantly affected by temperature amplitude, indicating that changes in life-history traits were driven mainly by shifts in mean trait values rather than by consistent among-individual variability. Notably, the CV in female fecundity did not change significantly with increasing amplitude, and longevity variation showed no increasing trend. Thus, egg-stage amplitude primarily caused a systematic reduction in mean longevity and fecundity rather than increasing inter-individual variability. In contrast, male longevity and its CV were not significantly affected by egg-stage temperature amplitude. This pronounced sex-specific sensitivity may reflect asymmetry in reproductive investment. Females require substantial energetic investment for ovarian development and egg production, making their longevity and reproductive performance more susceptible to early resource limitation or delayed effects of thermal stress. By comparison, males generally invest less energy in reproduction, and their longevity may therefore be less sensitive to early thermal history [40,41]. Consequently, suppression of female longevity and fecundity under wide egg-stage amplitudes is likely an important mechanism underlying subsequent declines in population performance. Such energy allocation conflicts may reflect fundamental life-history trade-offs between current reproduction and somatic maintenance [42].

### 4.4. Implications for Pest Forecasting and Management

Wide egg-stage temperature amplitudes weakened the population growth potential of *T*. *absoluta*. At 22 °C, generation time (*T*), intrinsic rate of increase (*r_m_*), net reproductive rate (*R*_0_), and finite rate of increase (*λ*) all declined with increasing amplitude. At 25 °C, the wide amplitude prolonged generation time relative to 25 ± 5 °C and sharply reduced fecundity, producing a 55.2% reduction in *R*_0_ relative to 25 ± 0 °C. Thus, models fitted only to constant-temperature or mean-temperature responses may overestimate population growth potential and misestimate phenological timing. Incorporating daily temperature variation can improve the realism of developmental and phenological predictions [43]. For *T. absoluta*, monitoring systems should therefore consider the thermal amplitude during the egg-exposure period as well as mean temperature. Field validation remains necessary because naturally varying humidity, host quality, behavioral thermoregulation, and multigenerational exposure may modify the laboratory responses observed here. In addition, because the experimental colony had been maintained for 10 generations under standardized laboratory conditions, the responses reported here represent those of a standardized laboratory population rather than a wild field population. Multigenerational laboratory rearing may lead to laboratory adaptation and divergence from field populations [44]. Further studies using field populations are therefore needed to determine whether similar responses to egg-stage thermal conditions occur under natural conditions.

## 5. Conclusions

The amplitude of the daily temperature at the egg stage induced both immediate and carry-over effects in the later life stage of *T*. *absoluta*. The direction or magnitude of several of these responses depended on mean temperature. The wide amplitude altered egg and larval development, reduced larval survival, shortened female longevity, decreased fecundity, and ultimately lowered population growth potential. These results show that the effects of the early thermal history can persist beyond the egg stage and affect demographic performance. Consequently, forecasting models based only on mean temperature may misestimate both phenological timing and outbreak potential. Incorporating egg-stage thermal history and daily temperature fluctuation into pest models could improve outbreak forecasting, risk assessment, and integrated pest management decisions under climate change.

## Data Availability

The data used to support the findings of this study are available from the corresponding author upon request.

## Supplementary Materials

The following supporting information can be downloaded at https://www.mdpi.com/article/10.3390/insects17101034/s1, Figure S1: Daily mean temperature and daily temperature amplitude recorded in the greenhouse; Figure S2: Target and recorded temperature regimes used in the experiment; Table S1: Effects of egg-stage temperature amplitude on developmental duration, longevity, fecundity, and coefficients of variation; Table S2: Weibull survival parameters under different temperature treatments; Table S3: 95% confidence intervals for coefficients of variation.
