# Supplementary material for: Egg-Stage Daily Temperature Amplitude Constrains Adult Fitness and Population Growth in Tuta absoluta (Meyrick)
Source: Insects. 2026 Oct 9;17(10):1034. doi: 10.3390/insects17101034 (PMC13705332; doi:10.3390/insects17101034)
Supplement: Supplementary file 1 [file insects-17-01034-s001.zip › insects-4606745-supplementary.pdf]

Supplementary Material

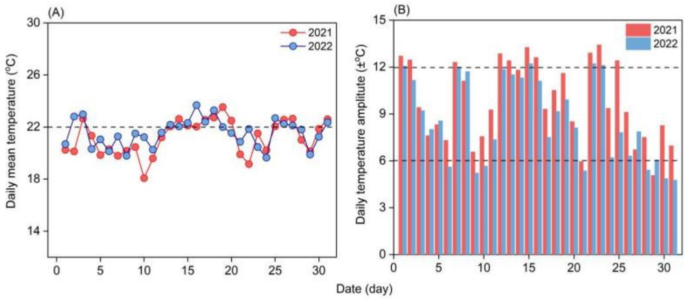

Fig. S1 Daily mean temperature (A) and daily temperature amplitude (B) in tomato greenhouses at Baiban Township during May 2021 and May 2022.

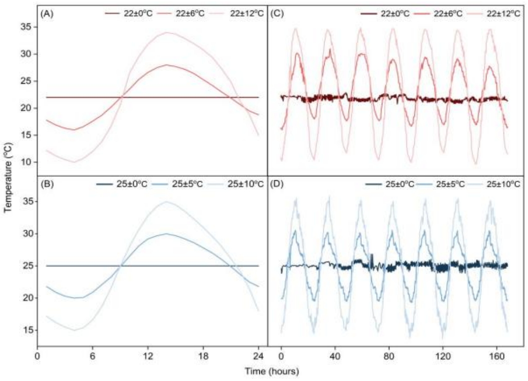

Fig. S2 Target temperature regimes (A, B) and temperatures recorded over seven consecutive days (C, D) in artificial climate chambers.

Table S1 Immediate and carry-over effects of egg-stage temperature amplitudes on life-history traits and their coefficients of variation (CVs) in *Tuta absoluta*.

Source		Trait						
Mean temperature (°C)	Temperature amplitude (± °C)	Egg development duration (d)	Larval development duration (d)	Female pupal development duration (d)	Male pupal development duration (d)	Female longevity (d)	Male longevity (d)	Fecundity (eggs/female)
22	0	4.93 ± 0.11	19.75 ± 0.28	9.23 ± 0.34	9.00 ± 0.28	15.27 ± 0.66	15.59 ± 1.02	84.68 ± 4.05
	6	4.55 ± 0.11	18.68 ± 0.33	8.39 ± 0.41	8.62 ± 0.35	11.38 ± 0.64	14.69 ± 1.25	62.08 ± 3.86
	12	4.07 ± 0.08	17.65 ± 0.43	8.38 ± 0.52	8.75 ± 0.46	11.25 ± 0.80	14.25 ± 1.55	54.75 ± 4.34
	χ^2	*43.08*	*17.628*	3.256	*0.764*	*23.496*	*0.625*	*29.017*
	P	*<0.001*	*<0.001*	*0.196*	*0.683*	*<0.001*	*0.732*	*<0.001*
25	0	5.27 ± 0.11	15.52 ± 0.19	8.14 ± 0.35	8.81 ± 0.34	18.19 ± 0.87	15.33 ± 0.81	129.43 ± 7.76
	5	3.88 ± 0.08	16.11 ± 0.21	8.56 ± 0.42	8.06 ± 0.35	14.56 ± 0.80	15.38 ± 0.39	102.00 ± 7.01
	10	4.48 ± 0.09	16.74 ± 0.22	8.94 ± 0.44	9.50 ± 0.42	13.00 ± 0.71	14.25 ± 0.86	72.13 ± 4.95
	χ^2	*109.063*	*17.72*	*2.044*	*7.075*	*22.874*	*1.061*	*41.165*
	P	*<0.001*	*<0.001*	*0.36*	*0.029*	*<0.001*	*0.588*	*<0.001*
Mean temperature (°C)	Temperature amplitude (± °C)	CV of egg developmental duration	CV of larval developmental duration	CV of female pupal developmental duration	CV of male pupal developmental duration	CV of female longevity	CV of male longevity	CV of fecundity
22	0	0.125 ± 0.008	0.092 ± 0.008	0.153 ± 0.088	0.138 ± 0.021	0.217 ± 0.034	0.215 ± 0.055	0.184 ± 0.032
	6	0.206 ± 0.014	0.088 ± 0.013	0.129 ± 0.032	0.112 ± 0.022	0.120 ± 0.025	0.216 ± 0.049	0.184 ± 0.041
	12	0.262 ± 0.015	0.092 ± 0.010	0.131 ± 0.041	0.115 ± 0.029	0.094 ± 0.027	0.185 ± 0.053	0.154 ± 0.044
	χ^2	*71.777*	*0.101*	*0.386*	*0.844*	9.665	3.239	*0.301*
	P	*<0.001*	*0.951*	0.824	*0.656*	*0.008*	*0.198*	*0.860*
25	0	0.157 ± 0.012	0.072 ± 0.008	0.152 ± 0.037	0.136 ± 0.023	0.136 ± 0.027	0.304 ± 0.038	0.200 ± 0.045
	5	0.178 ± 0.014	0.065 ± 0.008	0.189 ± 0.052	0.167 ± 0.033	0.211 ± 0.044	0.234 ± 0.051	0.241 ± 0.063
	10	0.185 ± 0.014	0.089 ± 0.008	0.130 ± 0.036	0.115 ± 0.022	0.219 ± 0.045	0.164 ± 0.036	0.228 ± 0.059
	χ^2	*2.695*	*4.766*	*0.927*	*1.817*	*3.81*	*1.362*	*0.319*
	P	*0.26*	*0.092*	*0.629*	*0.403*	*0.149*	*0.506*	*0.853*

Note: Data are presented as mean ± standard error (SE).

Table S2 Immediate and carry-over effects of egg-stage temperature amplitudes on Weibull mortality parameters across developmental stages of *Tuta absoluta*.

Trait	Source		Weibull scale parameter (*η*)		Weibull shape parameter (*β*)	
	Mean temperature (°C)	Temperature amplitude (± °C)	Mean ± SE	95% Confidence interval	Mean ± SE	95% Confidence interval
Egg survival	22	0	5.34 ± 0.09	5.167-5.519	6.01 ± 0.38	5.321-6.803
		6	5.25 ± 0.09	5.072-5.438	6.07 ± 0.45	5.250-7.010
		12	5.40 ± 0.11	5.190-5.620	5.22 ± 0.35	4.590-5.958
	25	0	5.90 ± 0.11	5.695-6.111	5.99 ± 0.42	5.215-6.870
		5	4.27 ± 0.08	4.113-4.430	5.79 ± 0.47	4.937-6.785
		10	5.09 ± 0.07	4.962-5.225	8.69 ± 0.68	7.446-10.738
Larval survival	22	0	20.79 ± 0.29	20.230-21.362	9.74 ± 0.90	8.131-11.672
		6	18.91 ± 0.44	18.058-19.793	9.76 ± 1.63	7.041-13.534
		12	18.65 ± 1.02	16.764-20.757	2.58 ± 0.30	2.069-3.227
	25	0	16.17 ± 0.19	15.807-16.551	11.42 ± 1.02	9.587-13.614
		5	16.78 ± 0.20	16.395-17.173	12.24 ± 1.22	10.076-14.873
		10	17.64 ± 0.29	17.075-18.222	8.76 ± 0.86	7.230-10.610
Female pupa survival	22	0	10.02 ± 0.43	9.213-10.899	5.23 ± 0.77	3.921-6.982
		6	8.47 ± 0.50	7.541-9.514	4.30 ± 0.79	2.997-6.178
		12	8.88 ± 0.36	8.196-9.618	9.04 ± 2.62	5.128-15.949
	25	0	8.78 ± 0.30	8.207-9.394	6.58 ± 1.07	4.795-9.040
		5	9.40 ± 0.49	8.490-10.403	5.03 ± 1.11	3.268-7.741
		10	9.56 ± 0.38	8.842-10.326	6.55 ± 1.43	4.273-10.034
Male pupa survival	22	0	9.62 ± 0.29	9.071-10.200	7.46 ± 1.24	5.387-10.338
		6	9.10 ± 0.29	8.544-9.691	9.11 ± 1.98	5.956-13.933
		12	9.24 ± 0.36	8.548-9.977	9.45 ± 2.68	5.417-16.494
	25	0	9.47 ± 0.33	8.843-10.734	6.56 ± 1.02	4.827-8.902
		5	8.81 ± 0.38	8.093-9.582	5.97 ± 1.03	4.252-8.384
		10	10.03 ± 0.28	9.502-10.583	9.55 ± 1.83	6.435-14.182

Table S3 Coefficients of variation (CVs) and their 95% bootstrap confidence intervals for life-history traits of *Tuta absoluta* under different amplitude temperature treatments.

Trait	Source		Mean ± SE	95% Confidence interval Lower limit	95% Confidence interval Upper limit
	Mean temperature (°C)	Temperature amplitude (± °C)			
Egg developmental duration	22	0	0.125 ± 0.008	0.1094	0.1426
		6	0.206 ± 0.014	0.1805	0.2351
		12	0.262 ± 0.015	0.2346	0.2919
	25	0	0.157 ± 0.012	0.1352	0.1816
		5	0.178 ± 0.014	0.1527	0.2079
		10	0.185 ± 0.014	0.1597	0.2152
larval developmental duration	22	0	0.092 ± 0.008	0.0780	0.1097
		6	0.088 ± 0.013	0.0652	0.1177
		12	0.092 ± 0.010	0.0742	0.1138
	25	0	0.072 ± 0.008	0.0573	0.0867
		5	0.065 ± 0.008	0.0486	0.0804
		10	0.089 ± 0.008	0.0730	0.1048
female pupal developmental duration	22	0	0.153 ± 0.088	0.1064	0.2216
		6	0.129 ± 0.032	0.0803	0.2087
		12	0.131 ± 0.041	0.0712	0.2406
	25	0	0.152 ± 0.037	0.0950	0.2436
		5	0.189 ± 0.052	0.1101	0.3238
		10	0.130 ± 0.036	0.0758	0.2230
male pupal developmental duration	22	0	0.138 ± 0.021	0.1028	0.186
		6	0.112 ± 0.022	0.0765	0.1653
		12	0.115 ± 0.029	0.0704	0.1881
	25	0	0.136 ± 0.023	0.0975	0.1903
		5	0.167 ± 0.033	0.1139	0.2452
		10	0.115 ± 0.022	0.0785	0.169
female longevity	22	0	0.217 ± 0.034	0.1588	0.2958
		6	0.120 ± 0.025	0.0802	0.1802
		12	0.094 ± 0.027	0.0559	0.1567
	25	0	0.136 ± 0.027	0.0957	0.1945
		5	0.211 ± 0.044	0.1404	0.3164
		10	0.219 ± 0.045	0.1458	0.3285
male longevity	22	0	0.215 ± 0.055	0.2044	0.4425

		6	0.216 ± 0.049	0.1395	0.3375
		12	0.185 ± 0.053	0.1055	0.3255
	25	0	0.304 ± 0.038	0.1406	0.3949
		5	0.234 ± 0.051	0.1528	0.3571
		10	0.164 ± 0.036	0.1073	0.2507
fecundity	22	0	0.184 ± 0.032	0.1308	0.2579
		6	0.184 ± 0.041	0.1181	0.2855
		12	0.154 ± 0.044	0.0879	0.2708
	25	0	0.200 ± 0.045	0.1281	0.3122
		5	0.241 ± 0.063	0.1447	0.4015
		10	0.228 ± 0.059	0.1370	0.3801
